# Formulation and In Vitro Evaluation of Pellets Containing Sulfasalazine and Caffeine to Verify Ileo-Colonic Drug Delivery

**DOI:** 10.3390/pharmaceutics13121985

**Published:** 2021-11-23

**Authors:** Annemarie Broesder, Said Y. Bircan, Anneko B. de Waard, Anko C. Eissens, Henderik W. Frijlink, Wouter L. J. Hinrichs

**Affiliations:** Department of Pharmaceutical Technology and Biopharmacy, Groningen Research Institute of Pharmacy, University of Groningen, Antonius Deusinglaan 1, 9713 AV Groningen, The Netherlands; a.broesder@rug.nl (A.B.); s.y.bircan@student.rug.nl (S.Y.B.); a.b.de.waard@student.rug.nl (A.B.d.W.); a.c.eissens@rug.nl (A.C.E.); h.w.frijlink@rug.nl (H.W.F.)

**Keywords:** ileo-colonic targeting, film coating, ColoPulse, extrusion–spheronization, pan coating, ethanol, sodium starch glycolate, croscarmellose sodium

## Abstract

The ColoPulse coating is a pH-dependent coating that can be used to target drug release to the ileo-colonic region. ColoPulse coated tablets and capsules have demonstrated their targeting capabilities in vivo in more than 100 volunteers and patients. However, so far the ColoPulse coating has not been used for multi-particulate pellet formulations. The sulfasalazine–caffeine method can be used to confirm ileo-colonic drug delivery in vivo. Caffeine serves as a release marker in this method, while sulfasalazine serves as a marker for colonic arrival. In this study, extrusion–spheronization was used to produce microcrystalline cellulose based pellets containing both caffeine and sulfasalazine. Dissolution tests revealed that a superdisintegrant, i.e., croscarmellose sodium or sodium starch glycolate, should be incorporated in the formulation to achieve acceptable release profiles for both sulfasalazine and caffeine. However, acceptable release profiles were only obtained when the pelletizing liquid consisted of ethanol/water 1/1 (*v/v*) but not with pure water. This phenomenon was ascribed to the differences in the degree of swelling of the superdisintegrant in the pelletizing liquid during the granulation process. The pellets were coated with the ColoPulse coating and showed the desired pH-dependent pulsatile release profile in vitro. In future clinical studies, ileo-colonic targeting should be verified.

## 1. Introduction

The ColoPulse coating is a pH-dependent coating that can be used to obtain ileo-colonic drug delivery with a pulsatile drug release profile [1]. The pH in the lumen of the gastrointestinal tract remains below pH 7.0 until the terminal ileum is reached where it exceeds this pH [2]. The ColoPulse coating is based on Eudragit S100, a pH-sensitive polymer, which dissolves at pH values above 7.0 [3]. The pulsatile drug delivery profile is obtained by incorporating a superdisintegrant in a non-percolating manner in the coating. This disintegrant ensures that the coating rapidly disintegrates once the pH threshold of pH-sensitive polymer is reached. A range of in vivo studies in healthy volunteers and Crohn’s disease patients have demonstrated that a reliable and effective release in the ileo-colonic region can be achieved with the ColoPulse technology [1,4,5,6]. In the first study, it was demonstrated that release was site-specific and occurred in the more distal part of the intestine [1]. Measurement of exhaled ^13^CO_2_, originating from ^13^C_6_-glucose in the capsule, was found to be directly related to the period between the capsule ingestion and the subsequent meal. Ingestion of food stimulates gastrointestinal motility, which caused the dosage form to pass the ileocecal junction. In a series of follow-up studies, ^13^C-urea was used as a marker to prove ileo-colonic release. When release occurs in higher parts of the gastrointestinal tract, the isotope will be excreted unchanged in the urine. But, when release occurs in the ileo-colonic region, the urea is metabolized by bacteria into ^13^CO_2_, which can be measured in the exhaled breath. The studies showed that the ColoPulse system released its contents in the ileo-colonic region in at least 88% of the 86 included cases [4,5,6]. In one of these studies, ColoPulse coated tablets were co-administered with the IntelliCap system [5]. The IntelliCap was used to measure the pH in the gastrointestinal tract. It was found that the ColoPulse tablets release their contents after a pH of 7.0 was reached in the distal ileum and colon. This study confirmed the pH-dependency of the ColoPulse coating. Furthermore, the pharmacokinetics of ColoPulse coated peppermint oil capsules for the treatment of irritable bowel syndrome were studied. Compared to an enteric coated formulation, the ColoPulse coated capsules showed a delayed peak plasma concentration and lag time [7].

Thus far, the ColoPulse coating has been applied on tablets or capsules, but it has never been applied on a multi-particulate system. For oral targeted drug delivery, the European Medicine Agency encourages the development of multiple-unit dosage forms, such as coated pellets, due to reduced risk of dose-dumping [6]. Next to a reduced risk in dose dumping, there are other advantages to multi-particulate systems, such as dosing flexibility, a reduced chance of gastrointestinal irritation, and less influence of the gastric residence time by the food status [7,8]. Further, pellets have a longer colonic residence time [9], which could be beneficial for slow release formulations targeting their release to the colon.

Extrusion–spheronization is a frequently used technique to produce pellets [10,11]. A plastic mass is produced by mixing a powder blend with a pelletizing liquid. Subsequently, this plastic mass is extruded to obtain rods. Finally, these rods are placed on a spinning friction plate where they are broken up and rounded off to obtain spherical pellets. Microcrystalline cellulose (MCC) is frequently used as a bulking agent due to its favorable spheronization properties [10]. MCC has a large surface area and high internal porosity and thus is capable of retaining water. When MCC is wetted, it forms a cohesive mass with good plasticity, because of the auto adhesion effect of wet MCC. However, a frequently encountered issue with MCC based pellet formulations is their slow release profile, due to the slow disintegration of the pellet formulation. Several strategies have been adopted to obtain a faster release profile, e.g., adding disintegrants to the formulation or partially substituting water with ethanol as the pelletizing liquid [10]. Disintegrants, such as sodium starch glycolate (SSG) and croscarmellose sodium (CCS), swell when they come into contact with water, which can enhance the disintegration of the pellets [12]. Furthermore, adding ethanol to the pelletizing liquid leads to a lower cohesive strength of the wetted mass and decreases the density of the final pellets formulation, thus enhancing water penetration and thereby the release of the drugs [10].

It is obvious that eventually the system needs to be tested in vivo. A suitable method to verify ileo-colonic drug delivery in vivo is the sulfasalazine–caffeine method [2]. In this method, sulfasalazine and caffeine are incorporated in the dosage form. By comparing plasma concentrations of both drugs, one can verify whether ileo-colonic drug delivery is obtained. Caffeine is used as the release marker since it is absorbed over the entire gastrointestinal membrane, while sulfasalazine is used as the marker that indicates colonic arrival. Sulfasalazine is not absorbed in the gastrointestinal tract but is metabolized by colonic bacteria into sulfapyridine. Sulfapyridine is subsequently absorbed into the systemic circulation. When both sulfapyridine and caffeine appear in the plasma at approximately the same time, ileo-colonic drug delivery is obtained. However, when caffeine already appears in the plasma a significant period of time before sulfapyridine appears, premature release of the drugs occurs. This method has been successfully applied in dogs for a time-dependent colon-targeted drug delivery system in which theophylline was used instead of caffeine [13]. Theophylline was substituted by caffeine because of the latter’s lower toxicity profile [14]. Sulfasalazine has been used in humans to obtain the orocecal transit time [15,16]. Both compounds can be detected in plasma at sufficiently low concentrations (0.1 µg/mL) [15,17]. To obtain more reliable results, both caffeine and sulfasalazine should be formulated into the same pellet formulation; when two different pellet formulations are used, one for caffeine and one for sulfasalazine, there is always a risk of segregation in the gastrointestinal tract, making the obtained data less reliable.

Therefore, in this study, we developed a ColoPulse coated pellet formulation containing both sulfasalazine and caffeine with a similar pulsatile release profile, which can be used in future clinical studies. Clinical studies in humans are recommended to confirm that this pH-dependent pellet system can be used in humans as a marker for colonic delivery [2], as the pH profile of individual dogs can differ from that of humans [18].

## 2. Materials and Methods

### 2.1. Materials

Sodium chloride, acetone, and ethanol 96% were obtained from BOOM B.V. (Meppel, The Netherlands). Hydrochloric acid, sodium hydroxide, and sodium dihydrogen phosphate dihydrate were obtained from MERCK (Darmstadt, Germany). Sulfasalazine (SAS) was obtained from Sigma Aldrich (St. Louis, MO, USA). CCS was obtained from FMC BioPolymer (Philadelphia, PA, USA). SSG and MCC PH102 were obtained from DFE Pharma (Goch, Germany). Gelatine Licaps^®^ size 0 capsules were a gift from Capsugel (Bornem, Belgium). Eudragit S100 was a gift from Evonik (Darmstadt, Germany). Macrogolum 6000 (PEG 6000) and talc ~350 Mesh were obtained from BUFA (IJsselstein, The Netherlands). Caffeine was obtained from Genfarma (Maarssen, The Netherlands). Demineralized water was used in all cases.

### 2.2. Extrusion–Spheronization

Various pellet formulations containing SAS and CAF in a weight ratio of 2.5:1 were prepared by extrusion–spheronization using a Multi Lab Caleva (Caleva Process Solutions Ltd., Sturminster Newton, UK), see Table 1. The weight ratio of 2.5:1 was based on pharmacokinetic calculations, which are given in Appendix A. First, the powder was mixed for 15 min at 70 rpm. After three minutes of mixing half of the pelletizing liquid was added at 5 mL/min. After 9 min of mixing the rest of the pelletizing liquid was added at 5 mL/min. Subsequently, the wet mass was extruded with a screw extruder at 70 rpm with a standard die plate of 1 mm. Lastly, the extrudate was spheronized at 2500 rpm for up to three minutes. The pellets were dried overnight on a tray at room temperature. The amount of pelletizing liquid was optimized based on the amount of fines (too low volume) and aggregates formed (too high volume) during spheronization. The coated pellets were stored at room temperature in closed glass vials before analysis and further processing.

### 2.3. ColoPulse Coating

To avoid massive segregation during the coating process, the pellets were sieved using a screen to obtain pellets with a size distribution of 0.85–1.25 mm before coating. The suspension to apply the ColoPulse coating contained 7 g Eudragit S100, 1 g PEG 6000, 3 g CCS and 2 g Talc in 150 mL acetone:water with a volume ratio of 49:1 [1]. An in-house-made mini rotating drum coater was used to coat the pellets with the ColoPulse coating. Five grams of the pellets were placed in a perforated glass drum, with a diameter of 8 cm, which was covered with nylon mesh to prevent loss of pellets. The drum was rotated at 32 rpm. The ColoPulse suspension was sprayed on the pellets with a 1 mm bore diameter nozzle (Schlick 970, Düsen-Schlick, Coburg, Germany) with a spray rate of 3.3 mL/min using a peristaltic pump (Minipuls 3, Gilson, Viliers le Bel, France). A heat gun was used to keep the temperature between 20–25 °C. The coated pellets were sieved using a screen to obtain pellets with a size range of 1.2–1.4 mm which had the optimal coating thickness determined with dissolution studies (data not shown). This particle size corresponded to an applied coating amount of approximately 40 mg/cm^2^ or a coating thickness of approximately 200 μm. The coated pellets were stored at room temperature in closed glass vials before analysis.

### 2.4. Particle Size Analysis

The particle size was analyzed with a QICPIC (SYMPATEC, Clausthal-Zellerfeld, Germany) connected to a GRADIS dispersion system with a drop height of 50 cm and an outlet width of 4 mm. The images were captured with an M8 lens (measuring range 20–6820 µm) at a frame rate of 75 Hz when an optical concentration of 0.2% was reached for up to 240 s. A VIBRI was used as the feeder and the VIBRI control was set at an optical concentration of 1.00% with an integral of 2.0. The WINDOX 5 software was used to compute the particle size and sphericity. The software calculated the sphericity with Equation (1), where *P_EQPC_* is the perimeter of the equivalent circle in μm, *P_real_* is the real perimeter in μm, and A is the surface area in μm^2^. The particle size was based on the cumulative volume distribution. For the particle size and sphericity, the 50th percentile was given, indicated by the *x*_50_ for the particle size and the s_50_ for the sphericity. The span was used as a measure for the width of the size distribution, see Equation (2) where *x*_90_ is the diameter in μm where 90% of the particles are smaller than this diameter, *x*_10_ is the diameter in μm where 10% of the particles are smaller, and *x*_50_ the diameter in μm where 50% of the particles are smaller.

(1) $$Sphericity=\frac{P_{EQPC}}{P_{real}}=\frac{2\;\sqrt{\pi \;\cdot A}}{P_{real}}$$

(2) $$Span=\frac{x_{90}-x_{10}}{x_{50}}$$

### 2.5. Tensile Strength Analysis

The crushing strength of the pellets was measured sixfold with a Dr. Schleuniger Pharmatron Model 6D tablet tester (Thun, Switzerland). The tensile strength in MPa was calculated with Equation (3) [19], where F is the crushing strength in N and *r* is the radius in mm.

(3) $$Tensile\;strength=\frac{F\;}{\pi \cdot r^{2}}\;$$

Statistical significance was determined using one-way ANOVA, *p* values < 0.05 were considered significant.

### 2.6. In Vitro Dissolution

The release of CAF and SAS was tested in a USP dissolution apparatus type 2 (Sotax AT 7, Sotax, Basel, Switzerland) with 1000 mL medium at 37 °C and a paddle speed of 50 rpm. The absorbance was measured every three minutes at 273 and 359 nm for CAF and SAS, respectively, for 1 h, with an in-line UV-spectrophotometer (Evolution 300 UV–VIS spectrophotometer, Thermo Fisher Scientific, Madison, WI, USA). The release and performance of the ColoPulse coated pellets were tested using the Gastro Intestinal Simulation System (GISS) media [20]. The GISS simulates the pH profile of the gastrointestinal tract in four distinct phases [20], i.e., phase I: the stomach (pH 1.2 ± 0.2; 2 h), phase II: proximal small intestine (pH 6.8 ± 0.2; 2 h), phase III: terminal ileum (pH 7.63 ± 0.12; 0.5 h), and phase IV: colon (pH 6.0 ± 0.25; 1.5 h). The release of the uncoated pellets (364 mg; 50 mg CAF and 125 mg SAS) was tested in 1000 mL GISS phase III and the ColoPulse coated pellets (364 mg; 16 mg CAF and 40 mg SAS) filled in Licaps size 0 capsules in GISS I-IV. The pellet weight of 364 mg was selected based on the filling capacity of the size 0 capsules. A sinker was used to place the capsules at the bottom of the dissolution vessel. In GISS phase II-IV sink conditions were present for both compounds [21,22].

### 2.7. Swelling Behavior Disintegrants

The swelling behavior of 5% *w/v* CCS and SSG in water and ethanol/water 1/1 (*v/v*) was evaluated with a camera against a black background. The decrease in swelling of CCS and SSG in water/ethanol compared to water was determined by linear measurements using ndp.view2 (version 2.9.25; Hamamatsu, Japan), see Equation (4) where *px* is pixels.

(4) $$Decrease\;in\;swelling\;\left(\%\right)=\frac{px_{water}-px_{EtOH}}{px_{water}}\;\cdot 100\%$$

## 3. Results and Discussion

### 3.1. Uncoated Pellets

**Figure 1:**
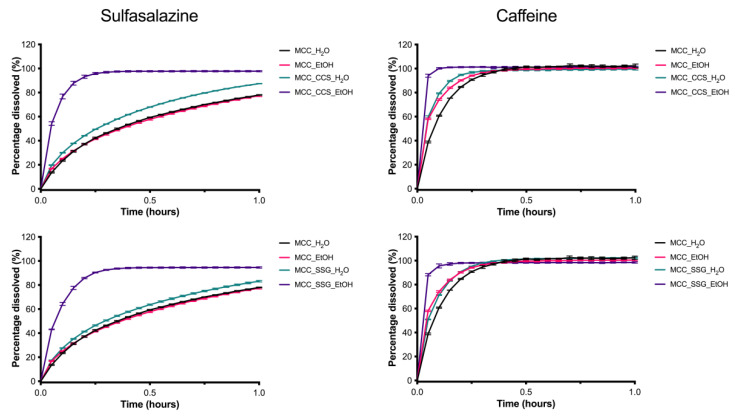
Influence of excipients and pelletizing liquid on the release profile of SAS (**left**) and CAF (**right**) in 1000 mL GISS phase III (mean ± SD, n = 3).

In Table 2, an overview of the characteristics of the different pellet formulations is provided. All formulations resulted in spherical pellets with a particle size around 1 mm. The MCC_H_2_O pellets provided sufficient release of CAF, ≥80% within 30 min, but this was not the case for SAS. This difference in dissolution can be explained by CAF’s higher water solubility compared to SAS [21,22], i.e., 21 mg/mL and 4–7 mg/mL, respectively, as well as by the fact that the pellets did not fully disintegrate. In Figure 1, it can be seen that replacing pure water with ethanol/water 1/1 (*v/v*) as the pelletizing liquid, i.e., the MCC_EtOH formulation, did not improve the dissolution profile of SAS. The CAF release on the other hand was marginally improved. To induce rapid disintegration of the pellets the superdisintegrants CCS and SSG were added to the formulation. CCS and SSG can improve the dissolution of poorly or slightly soluble drugs, such as SAS, by their strong swelling behavior in water, which can result in the disintegration of the formulation [12]. However, when water was used as the pelletizing liquid it was visually observed that the pellet formulations MCC_CCS_ H_2_O and MCC_SSG_ H_2_O, did not substantially disintegrate during the dissolution test. This is also indicated by a slightly improved release of SAS (Figure 1). In contrast, when ethanol/water 1/1 (*v/v*) was used as the pelletizing liquid, it was visually observed that the pellet formulations MCC_CCS_EtOH and MCC_SSG_EtOH immediately disintegrated in the dissolution medium. The rapid disintegration of the pellets resulted in over 80% release of the SAS within 12 min (Table 2 and Figure 1) for both superdisintegrants. The CCS containing pellet formulation, i.e., MCC_CCS_EtOH, gave a slightly faster release than the SSG containing formulation, i.e., MCC_SSG_EtOH. The tensile strength of the pellets indicated that the mechanical robustness of the pellets was not responsible for the increased dissolution rate of the MCC_SSG_EtOH and MCC_CCS_EtOH formulations.

**Figure A2:**
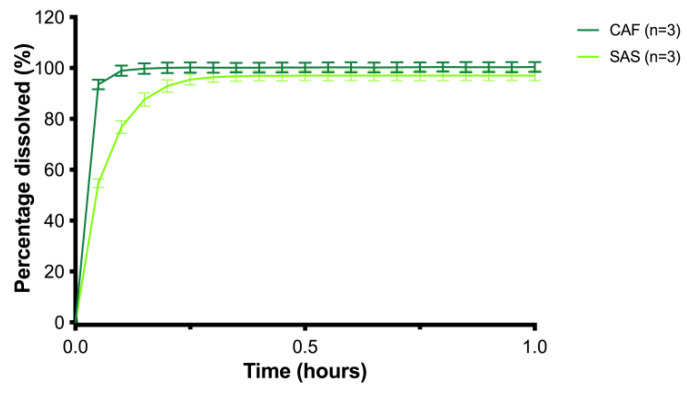
Reproducibility of SAS and CAF release in 1000 mL GISS phase III from three different batches of MCC_CCS4_EtOH (mean ± SD, n = 3 batches).

The production process of the MCC_CCS_EtOH pellet formulation was robust, indicated by the reproducible in vitro release (Figure A2 and Table A1) and particle size, particle size distribution, sphericity, and tensile strength (Table A1).

**Figure 2:**
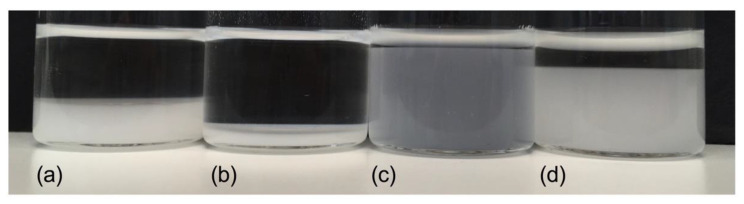
Swelling behavior of CCS and SSG in water and ethanol/water 1/1 (*v/v*). (**a**) CCS in ethanol/water 1/1 (*v/v*); (**b**) SSG in ethanol/water 1/1 (*v/v*); (**c**) CCS in water; (**d**) SSG in water.

The swelling behavior of CCS and SSG in water and ethanol/water 1/1 (*v/v*) was investigated to elucidate the effect of the pelletizing liquid on pellet disintegration. As can be seen in Figure 2, both CCS and SSG swell much less in ethanol/water 1/1 (*v/v*) than in pure water. The decrease in swelling in ethanol/water compared to water for CCS was 63% and for SSG 86%, indicating that the effect of ethanol was more pronounced for SSG. The larger impact on the swelling behavior of SSG might be explained by the high hydration capacity of SSG compared to CCS, with 18.3 and 12.1 g water/g polymer, respectively [23]. Even though the swelling behavior of SSG was more impacted, incorporation of CCS in the pellets produced slightly higher dissolution rates (Figure 1). This might be due to the higher swelling pressure generated by CCS than SSG, resulting in faster disintegration [23]. During the production process, the addition of superdisintegrants required a higher amount of water as the pelletizing liquid to reduce the amount of fines produced (data not shown). In contrast, when ethanol/water 1/1 (*v/v*) was used as the pelletizing liquid, the difference in the amount needed was less pronounced. This was expected in light of their working mechanism [12] and is supported by the results shown in Figure 2.

**Figure 3:**
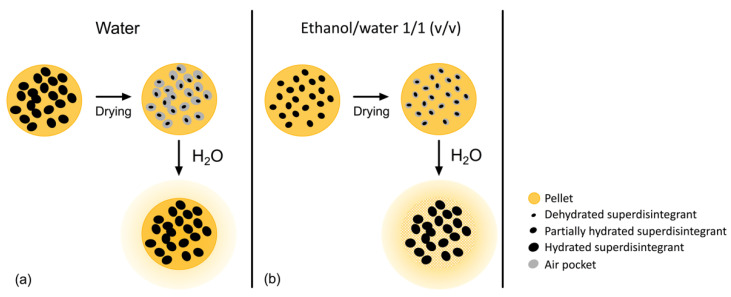
Schematic representation of the influence of the pelletizing liquid during the production process on the disintegration behavior of the pellet in aqueous dissolution media. (**a**) Water as a pelletizing liquid leads to air pockets in the dried formulation hampering disintegration during dissolution; (**b**) ethanol/water 1/1 (*v/v*) as a pelletizing liquid leads to smaller air pockets in the dried formulation enabling disintegration during dissolution.

In a study by Bisharat et al., the effects of ethanol on superdisintegrants was investigated [24]. They found that ethanol/water 1/1.5 (*v/v*) compared to pure water reduced the degree of swelling of tablets made entirely from superdisintegrants. Furthermore, they showed that tablets containing 4% superdisintegrants showed longer disintegration times in ethanol/water 1/1.5 (*v/v*) than in pure water. They also found that the reduced swelling of the superdisintegrants led to higher tablet disintegration times in ethanol/water 1/1.5 (*v/v*) than in pure water [24]. In our study, an ethanol/water mixture was used in the production process rather than in the media used for disintegration testing. However, based on our findings and those of Bisharat et al., we propose the following mechanism to explain the fast disintegration of the pellets when superdisintegrants are incorporated in the pellet formulation using ethanol/water mixtures as the pelletizing liquid. When pure water is used, the superdisintegrant particles will substantially swell during granulation. During subsequent drying, the superdisintegrant particles are dehydrated, and as a result, they will shrink, resulting in air pockets in the pellet. Therefore, when these pellets are subjected to an aqueous dissolution fluid, the superdisintegrant particles will swell to the same size as during granulation, exactly filling up the air pockets (Figure 3a). As a consequence, they will not be able to break up the pellets. However, when an ethanol/water mixture is used as pelletizing liquid, the superdisintegrant particles will swell less, resulting in smaller air pockets during drying. When these pellets are subjected to the aqueous dissolution fluid, the superdisintegrant particles will swell to a larger size as during granulation (Figure 3b). As a result, they will exert a substantial pressure on the pellet formulation resulting in rapid disintegration. The significantly reduced tensile strength of the MCC_CCS_H_2_O and MCC_SSG_H_2_O formulations compared to MCC_H_2_O underline the formation of the air pockets when water is used as the pelletizing liquid. The ColoPulse coating relies on a similar mechanism. The CCS is only very limited hydrated during the coating process since it is suspended in acetone:water with a volume ratio of 49:1. During the drying process, the CCS particles are surrounded by the other coating components. However, when the coating is placed in a solution where water can penetrate the coating, i.e., an aqueous solution with a pH > 7.0, the CCS particles are exposed resulting in swelling and subsequent disintegration of the coating layer.

Furthermore, Souto et al. incorporated superdisintegrants in MCC pellets using ethanol/water mixtures as pelletizing liquid [25]. In contrast to our results, they found that CCS as well as SSG were unable to induce fast disintegration of pellet formulations. On the other hand, these superdisintegrants did promote the dissolution of the poorly water-soluble drug. The improved dissolution behavior was ascribed to increased micropore volumes, which is contradictory to our proposed mechanism. Contradictory results can also be found in studies where pure water was used as the pelletizing liquid. Some studies showed that CCS and SSG can improve the disintegration of pellets [26,27], while others did not find such an improvement [25,28]. These contradictory results might be due to differences in formulation compositions and process parameters. Apparently, the effects of incorporating superdisintegrants in MCC pellets and the composition of the pelletizing liquid on the in vitro release characteristics should be investigated case by case.

Nevertheless, the MCC_CCS_EtOH pellet formulation produced the best in vitro release among the different formulations and was therefore selected to be coated with the ColoPulse coating.

### 3.2. ColoPulse Coated Pellets

**Figure 4:**
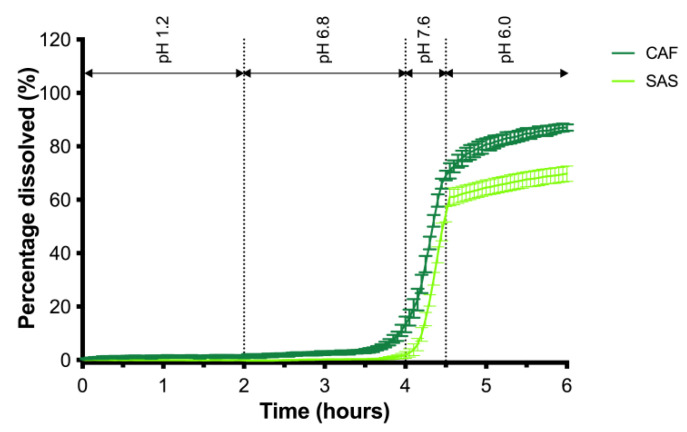
Release of SAS and CAF release in GISS I-IV from ColoPulse coated MCC_CCS_EtOH pellets (mean ± SD, n = 3).

The sieved ColoPulse coated pellets had a particle size, *x*_50_, of 1697.1 ± 4.1 μm, a sphericity of 0.918 ± 0.000, and a span of 0.40. In Figure 4 the release of the ColoPulse coated pellets in the GISS is given. Both SAS and CAF show a pulsatile release in phase III of the GISS, i.e., the simulated terminal ileum. The dissolution rate of SAS decreased in phase IV compared to phase III. The solubility of SAS is pH-dependent and it has a solubility of 4–7 mg/mL at pH 7.4, 2–3 mg/mL at pH 6.8, and 0.2–0.4 mg/mL at pH 5.8 [22]. Thus, the decreased dissolution rate can be explained by the lower solubility of SAS in GISS phase IV than in GISS phase III, even though sink conditions were maintained in phase IV of the dissolution test. At the end of the dissolution test, 70% of SAS and 87% of CAF were released. The lower release of SAS can be explained by the fact that SAS has a lower solubility than CAF [21]. However, the release profiles are sufficiently similar to proceed to in vivo studies. The majority of the dosage of both compounds is released in the simulated terminal ileum, i.e., phase III. The start of the drug release differs slightly. Ten percent of drug release was obtained after 3 h and 57 min for CAF and 4 h and 15 min for SAS. As the sampling interval in in vivo studies will be lower than in the dissolution test in this study, this difference of 18 min will be negligible. Furthermore, ten percent of drug release is close to the lower limit of quantification of 0.1 µg/mL of both compounds [15,17].

It should be mentioned that a pan coater was used for coating the pellets due to the small batch size. During pan coating, the pellets tumble over each other, which requires a dryer coating process to prevent aggregation of the pellets. The dryer coat process increases the risk of poorer coat quality due to pore formation. Next to this, segregation of pellets will occur during coating, resulting in an uneven coating thickness between the different pellets. To select the pellets with an optimal coating thickness the pellets were sieved and the dissolution of the obtained sieve fractions was evaluated (data not shown). The different sieve fractions gave pellets with a different particle size and as a result a different coating thickness. The 1.2–1.4 mm sieve fraction gave the desired release profile in the GISS, i.e., a pulsatile release in phase III, and therefore had the optimal coating thickness. Pellets with a smaller size and thus a thinner coating resulted in premature release in phase II of the GISS and pellets with a larger size and thus a thicker coating did not show a pulsatile release profile in phase III (data not shown). To circumvent these issues a fluidized bed coater would be more suitable and is recommended for upscaling. In fluid bed coaters, the pellets are fluidized by air and are less prone to aggregation and segregation [3]. This allows for a wetter coating process, which leads to better spreading of the coating solution preventing pore formation and twinning. Next to this fluidized bed, coaters have higher efficiency, leading to shorter process times.

## 4. Conclusions

In this study, we developed a multiple-unit drug delivery system that was coated with the pH-dependent ColoPulse coating. To verify ileo-colonic drug delivery in a clinical study a combination of SAS and CAF can be used. MCC based pellet formulations containing both SAS and CAF were produced by extrusion–spheronization. Pellets produced with water as the pelletizing liquid were, however, unable to generate an immediate release of SAS. Incorporating the superdisintegrants in the formulation along with the use of ethanol/water 1/1 (*v/v*) as a pelletizing liquid promoted the disintegration of the pellet formulation during dissolution and increased the dissolution rate. As a result, 80% release was obtained within 15 min for both CAF and SAS. We hypothesize that ethanol/water 1/1 (*v/v*) prevents substantial swelling of the superdisintegrant during the production process, thereby reducing air pocket formation during the drying process. As a result, the superdisintegrant can disintegrate the pellet formulation when immersed in water. Subsequent coating of the fast disintegrating pellet formulation with the ColoPulse coating resulted in a pulsatile release of CAF and SAS in the simulated terminal ileum. The ileo-colonic targeting of the formulation should be verified in future clinical studies.

## Figures and Tables

**Table 1:** Composition of the pellet formulations.

Formulations	Composition (g)					Pelletizing Liquid (mL)	
	SAS	CAF	MCC	CCS	SSG	Water	Ethanol/Water 1/1 (*v/v*)
MCC_H2O	3.571	1.429	5			7	
MCC_EtOH	3.571	1.429	5				7.75
MCC_CCS4_H2O	3.571	1.429	5	0.4		9	
MCC_SSG4_H2O	3.571	1.429	5		0.4	9	
MCC_CCS4_EtOH	3.571	1.429	5	0.4			8
MCC_SSG4_EtOH	3.571	1.429	5		0.4		8

**Table 2:** Characteristics of the pellet formulations (mean ± SD, n = 3, except tensile strength were n = 6).

Formulation	Release at t = 30 min (%)		Time at 80% Release (min)		Particle Size x_50_ (μm)	Span	Sphericity s_50_	Tensile Strength (MPa)
	SAS	CAF	SAS	CAF					
MCC_H_2_O	59.3 ± 0.4	101.5 ± 0.7	66	12	1126.2 ± 0.2	0.25	0.943 ± 0.000	14.0 ± 3.5
MCC_EtOH	57.8 ± 0.7	99.6 ± 0.7	66	9	1094.6 ± 2.2	0.38	0.938 ± 0.000	8.2 ± 1.1
MCC_CCS_H_2_O	67.9 ± 0.2	98.8 ± 0.8	48	9	982.6± 2.6	0.60	0.920 ± 0.001	8.4 ± 1.3 *
MCC_SSG_H_2_O	63.7 ± 0.6	101.4 ± 0.4	57	12	1137.9 ± 15.5	0.80	0.931 ± 0.001	8.4 ± 1.4 *
MCC_CCS_EtOH	97.7 ± 0.6	101.1 ± 0.7	9	3	1082.7 ± 2.4	0.39	0.938 ± 0.000	9.4 ± 1.8
MCC_SSG_EtOH	94.4 ± 0.6	98.4 ± 0.6	12	6	1073.8 ± 1.0	0.39	0.936 ± 0.000	11.0 ± 1.1 **

* Significantly lower than MCC_H20 (*p* < 0.01). ** significantly higher than MCC_EtOH (*p* < 0.01).

**Figure A1:**
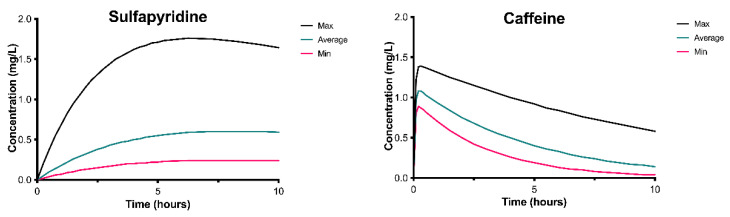
Plasma concentration of sulfapyridine and caffeine after oral administration of 125 mg sulfasalazine and 50 mg caffeine to a person with a body weight of 70 kg. The maximum, average, and minimum expected plasma concentration profiles are given based on pharmacokinetic parameters obtained from the literature [30,31].

## Appendix A

Equation (A1) gives the plasma concentration after a single oral dose [29], where *C* is the concentration in mg/L, *F* is the biological availability, *D* is the dose in mg, *k_a_* is the absorption rate constant in h^−1^, *V_d_* is the apparent volume of distribution in L, *k_e_* is the elimination rate constant in h^−1^ and *t* is the time in h.

(A1) $$C=\frac{F\;\cdot \;D\;\cdot \;k_{a}}{Vd\;\cdot \;\left(k_{a}-k_{e}\right)}\;\cdot \left(e^{-k_{e}\;\cdot \;t}-e^{-k_{a}\;\cdot \;t}\right)$$

## Appendix B

**Table A1:** Characteristics of different batches of the MCC_CCS4_EtOH pellet formulation.

Batch	Release (%) at t = 30 min		Time (Min) ≥ 80% Release		Particle Size x_50_ (μm)	Span	Sphericity s_50_	Tensile Strength (MPa)
	SAS	CAF	SAS	CAF				
MCC_CCS4_EtOH_1	94.8 ± 0.4	98.0 ± 0.8	9	3	1088.3 ± 2.9	0.38	0.937 ± 0.000	10.5 ± 0.9
MCC_CCS4_EtOH_2	98.5 ± 0.6	101.5 ± 1.2	9	3	1073.1 ± 1.2	0.42	0.938 ± 0.000	10.8 ± 3.6
MCC_CCS4_EtOH_3	97.7 ± 0.6	101.1 ± 0.7	9	3	1082.7 ± 2.4	0.39	0.938 ± 0.000	9.4 ± 1.8
